# Conduit Bleeding Unmasks Isolated Distal Ureter Recurrence After Radical Cystectomy: Lessons in Laparoscopic Nephroureterectomy and Surveillance

**DOI:** 10.7759/cureus.88821

**Published:** 2025-07-26

**Authors:** Roshan Yedulla Reddy, Rajan Ravichandran, Rubina Singh, Chandru T, Natarajan Kumaresan

**Affiliations:** 1 Urology, Sri Ramachandra Institute of Higher Education and Research, Chennai, IND

**Keywords:** adjuvant chemotherapy, conduit bleeding, radical cystectomy, upper urinary tract urothelial carcinoma, uretero-enteric anastomosis

## Abstract

Recurrent upper-tract urothelial carcinoma (UTUC) following radical cystectomy is an uncommon but clinically significant occurrence. We present the case of a 48-year-old man with a history of muscle-invasive bladder cancer (pT2N0M0) treated with radical cystectomy and Bricker ileal conduit diversion. He remained recurrence-free on structured surveillance for 42 months before developing isolated, painless stomal bleeding without associated systemic symptoms. Cross-sectional imaging revealed a 1.6 cm enhancing mass at the left uretero-enteric anastomosis with mild proximal hydroureteronephrosis, and PET-CT confirmed localized hypermetabolic activity without nodal or distant spread. The patient underwent laparoscopic radical nephroureterectomy with en-bloc excision of the distal ureter and limited conduit revision. Histopathology confirmed high-grade invasive UTUC (pT2) with lympho-vascular invasion, negative surgical margins, and a negative hilar node. He subsequently received four cycles of adjuvant gemcitabine-cisplatin (GC) chemotherapy. 18 months postoperatively, the patient remains asymptomatic with preserved renal function and no evidence of radiological recurrence. This case highlights the importance of considering upper-tract recurrence in the differential diagnosis of conduit bleeding. It supports timely cross-sectional imaging and oncologic resection as key steps in achieving long-term disease control.

## Introduction

Upper-tract urothelial carcinoma (UTUC) occurring after radical cystectomy and urinary diversion is an uncommon but clinically important entity, affecting approximately 1% to 6% of post-cystectomy patients [1]. Recurrence of UTUC often presents at more advanced stages compared to de novo tumors and are associated with reduced cancer-specific survival [2]. Proposed risk factors include positive distal ureteric margins, pre-existing carcinoma in situ, and chronic upper-tract obstruction [3]. The phenomenon of field cancerization is widely accepted in urothelial malignancies, supporting the concept of pan-urothelial susceptibility in genetically primed epithelium exposed to carcinogens [4].

Advances in molecular profiling have revealed that a significant proportion of UTUCs harbor FGFR3 mutations and exhibit a luminal-papillary phenotype, offering opportunities for future targeted therapies [5]. Current surveillance guidelines recommend routine upper-tract imaging following cystectomy, typically using contrast-enhanced CT urography due to its high sensitivity [6]. However, concerns remain regarding cumulative radiation exposure and cost-effectiveness [7]. Magnetic resonance urography has emerged as a viable nephrotoxicity-sparing alternative, particularly in patients with urinary diversions [8]. Furthermore, liquid biopsy using circulating tumor DNA is gaining traction as a non-invasive strategy for earlier detection of recurrence [9].

Despite established protocols, upper-tract recurrence may still be overlooked, especially when symptoms are subtle or non-specific. Isolated conduit bleeding is often misattributed to benign causes such as stomal trauma or infection. This case of recurrence that arose 42 months after cystectomy at the left uretero-enteric anastomosis highlights the importance of considering upper-tract recurrence in patients with new-onset bleeding from an ileal conduit. It emphasizes the role of timely imaging and surgical intervention in achieving favorable oncological outcomes. 

## Case presentation

**Figure 1:**
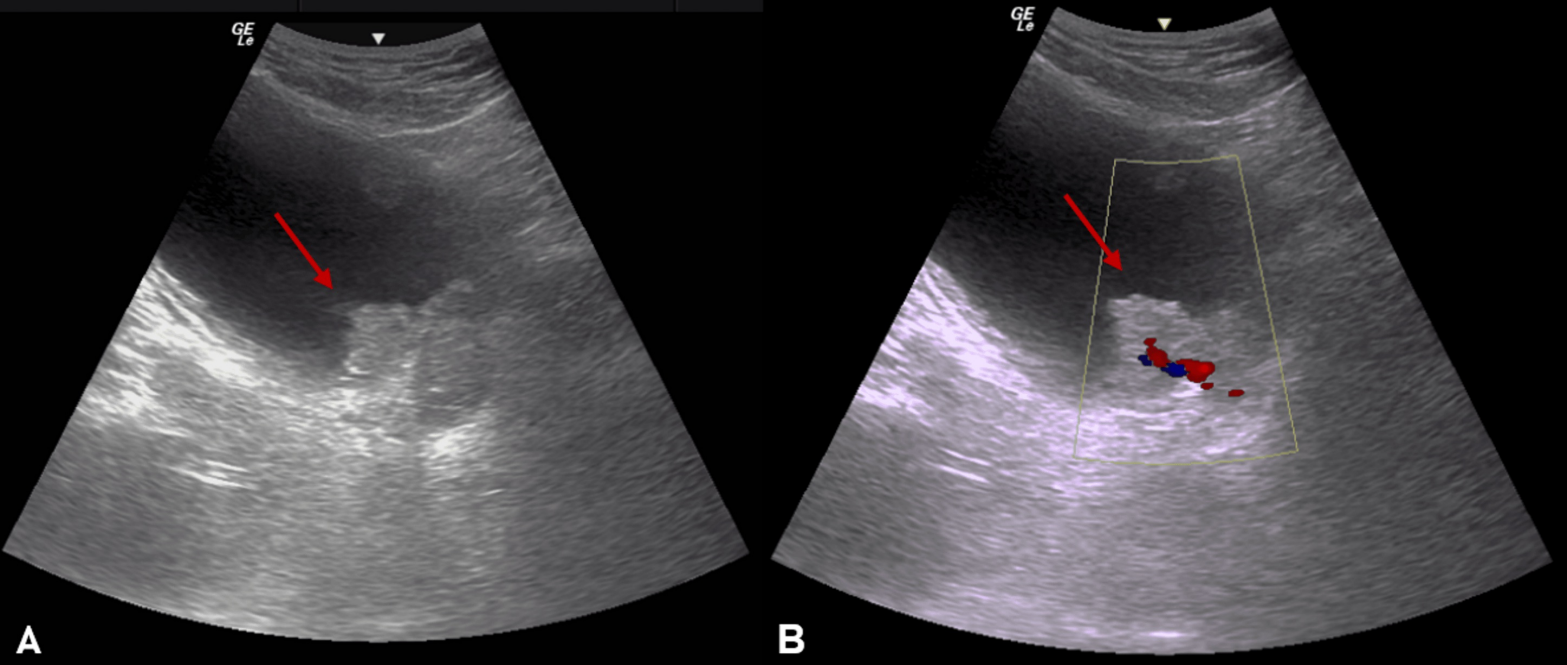
Ultrasonography identifying a solitary papillary lesion (A) Gray-scale sagittal ultrasound demonstrates a well-defined hyperechoic intravesical mass arising from the left posterior wall (red arrow); (B) Color Doppler confirms internal vascular flow within the lesion (red arrow).

**Figure 2:**
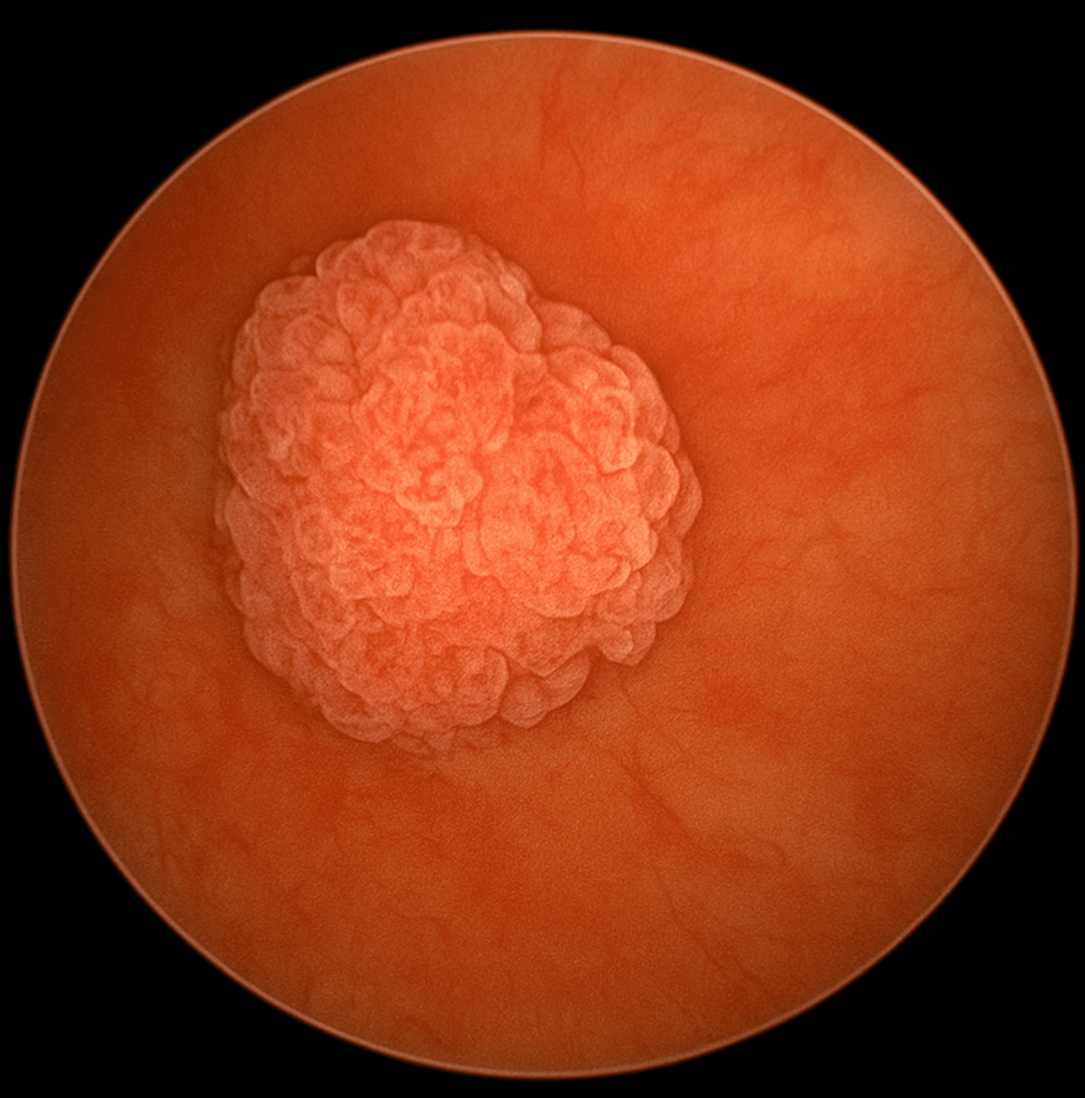
Cystoscopic view of left posterolateral wall bladder tumor Rigid cystoscopy showing a solitary 3.5 cm papillary bladder tumor originating from the left posterolateral wall. The lesion appears exophytic, lobulated, and vascular, consistent with high-grade urothelial carcinoma. The surrounding mucosa appears normal without satellite lesions or evidence of diffuse carcinoma in situ.

**Figure 3:**
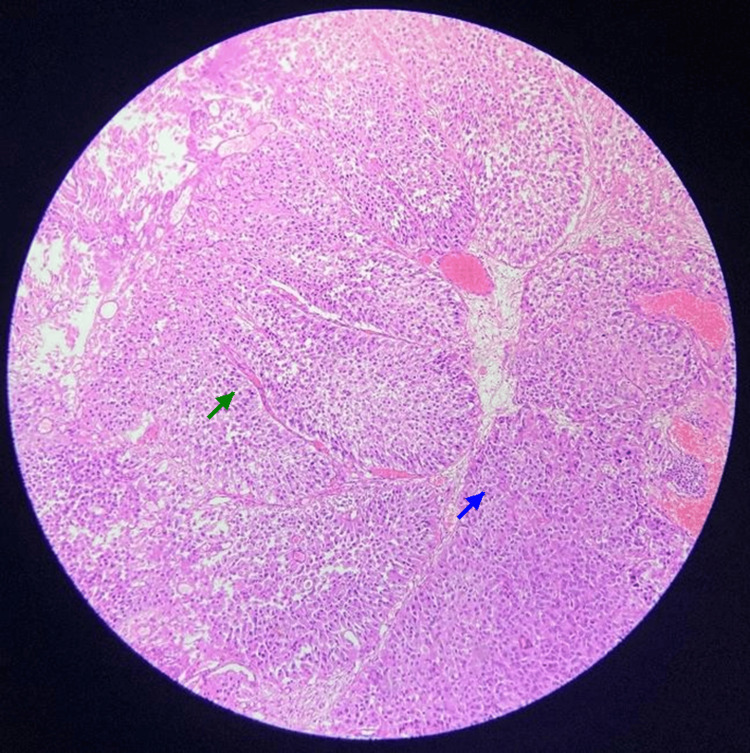
Histopathological examination of resected bladder tumor with H&E H&E-stained section (×100 magnification) showing high-grade urothelial carcinoma. Large, pleomorphic nuclei and prominent nucleoli (blue arrow) with brisk mitotic activity are seen. Papillary architecture and focal areas of invasion into the lamina propria (green arrow) are evident. No evidence of muscularis propria invasion is seen in this field. H&E:  Hematoxylin and eosin

A 48-year-old ex-smoker (15 pack-years) with a body mass index of 26 kg/m² presented with five days of gross, painless haematuria. Ultrasonography (Figure 1) and cystoscopy (Figure 2) identified a solitary papillary lesion measuring 3.5 × 2.8 cm on the left posterior bladder wall. Contrast-enhanced CT confirmed a localized bladder tumor without upper-tract involvement. Transurethral resection of the bladder tumor was performed, and histopathology showed high-grade muscle-invasive urothelial carcinoma staged as T2N0M0 (Figure 3).

The patient subsequently underwent open radical cystectomy with extended pelvic lymphadenectomy and Bricker end-to-side uretero-ileal anastomosis. Distal ureteric frozen sections were negative, and postoperative recovery was uneventful with a baseline creatinine of 1.1 mg/dL.

**Figure 4:**
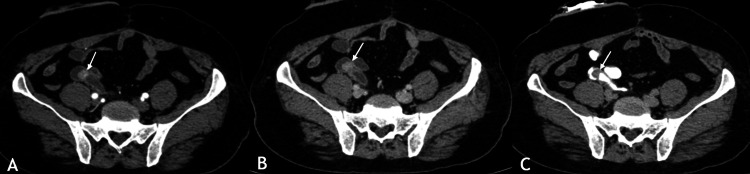
Triple-phase axial contrast-enhanced CT images demonstrate a distal left ureteric lesion at the uretero-enteric anastomosis (white arrows) Arterial phase (A) shows an avidly enhancing soft tissue lesion (white arrow) at the distal left ureter. Venous phase (B) reveals persistent enhancement of the lesion with improved contrast between the mass and adjacent conduit loops. The delayed excretory phase (C) shows continued enhancement of the lesion along with upstream mild left-sided hydroureteronephrosis, indicating partial functional obstruction. No regional lymphadenopathy or adjacent fat stranding was observed across phases.

**Figure 5:**
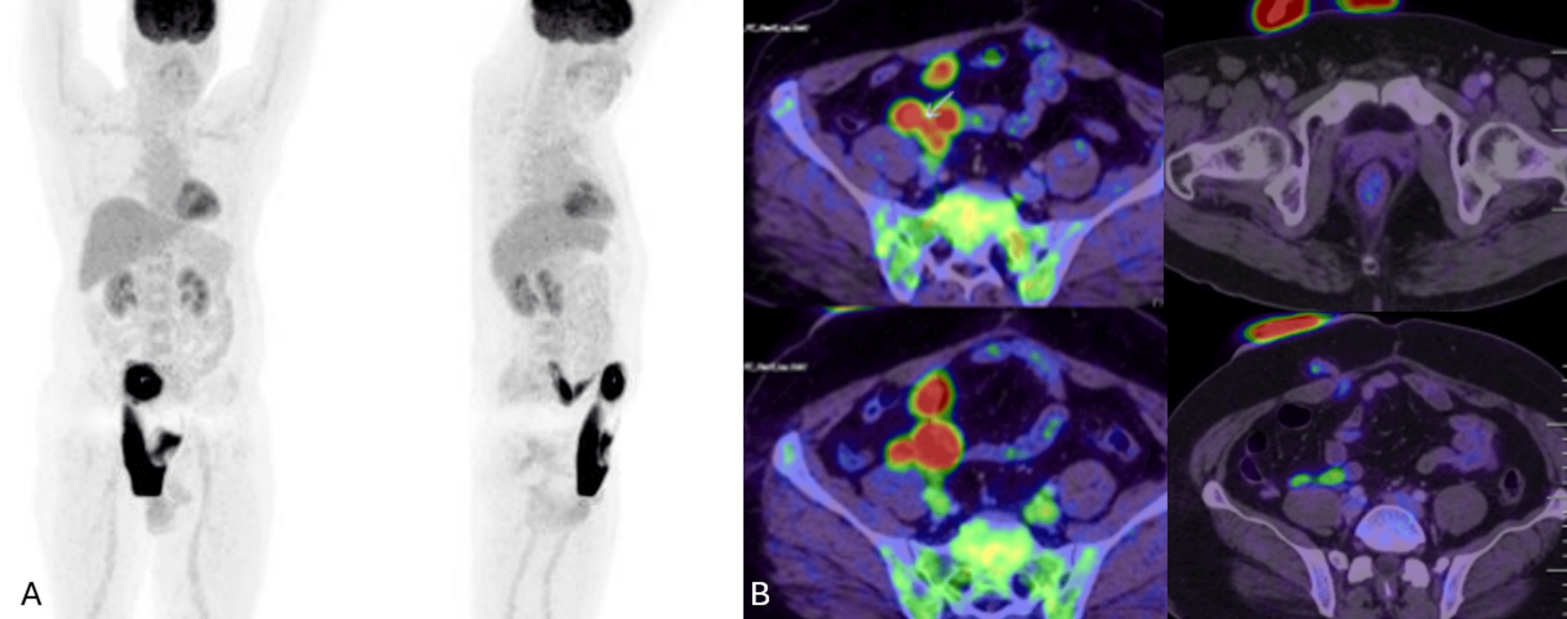
18F-FDG PET-CT evaluation of the recurrent lesion (A) Whole-body coronal and sagittal MIP images showing focal radiotracer uptake corresponding to the uretero-enteric junction; (B) Fused PET-CT axial images (top left and bottom left) reveal intense FDG uptake (SUV_max_ 7.8) at the left distal ureteric mass, with corresponding non-avid CT images (top and bottom right) showing the soft tissue lesion without nodal or distant metastatic involvement. FDG: Fluorodeoxyglucose; MIP: Maximum intensity projection; SUV_max_: Maximum standardized uptake value

Semi-annual follow-up with cytology, ultrasound, and annual CT urography remained unremarkable for 42 months. He then reported three episodes of painless bleeding from the stoma, without flank pain, infection, or conduit dysfunction. Laboratory evaluation revealed a hemoglobin level of 12.8 g/dL, normal coagulation parameters, and stable renal function (Table 1). Contrast-enhanced CT demonstrated a 1.6 cm avidly enhancing exophytic lesion at the distal left ureter just proximal to the uretero-ileal anastomosis, accompanied by mild upstream hydroureteronephrosis (Figure 4). 18F-fluorodeoxyglucose (FDG) PET-CT confirmed intense uptake (maximum standardized uptake value (SUV_max_) 7.8) without nodal or distant metastases (Figure 5).

**Table 1:** Baseline laboratory results at presentation Reference ranges from Mayo Clinic Laboratories [10] eGFR: Estimated glomerular filtration rate; INR: International Normalized Ratio

Parameters	Patient Value	Units	Reference Range
Hemoglobin	12.8	g/dL	13.5–17.5 (M) | 12.0–15.5 (F)
White-blood-cell count	7.2	×10⁹/L	4.0–11.0
Platelet count	265	×10⁹/L	150–450
Serum creatinine	1.1	mg/dL	0.7–1.3 (M) | 0.6–1.1 (F)
eGFR	76	mL/min/1.73 m²	>60
Prothrombin time	12.5	seconds	11.0–13.5
INR	1	—	0.8–1.2

Management approach

After multidisciplinary discussion, the patient consented to laparoscopic radical nephroureterectomy with en-bloc excision of the distal ureteric segment and limited conduit revision, rather than complete re-diversion. The surgical technique mirrored a previously described approach [11].

Following standard bowel preparation, the patient was positioned supine with a 60° left‑flank elevation. Five trans‑peritoneal ports were placed: a 12‑mm supra‑umbilical camera port, three 8‑mm working ports, and one 5‑mm assistant port. Adhesiolysis was performed sharply and with LigaSure Vessel Sealer (Medtronic, Ireland). The ureter was mobilized in a retro-colic plane, with Hem-o-Lok clips (Teleflex Medical, USA) controlling gonadal and ureteric vessels before stapling a distal cuff with an Endo-GIA (Medtronic, Ireland) permitting en‑bloc extraction of the kidney, ureter, and conduit segment.

Conduit continuity was re‑established with an end‑to‑side Wallace anastomosis using 4‑0 PDS absorbable suture (Ethicon (Johnson & Johnson), USA) over a 6‑Fr externalized single‑J stent; intraluminal methylene‑blue confirmed a watertight seal. Total operative time was 190 min and estimated blood loss 220 ml.

**Figure 6:**
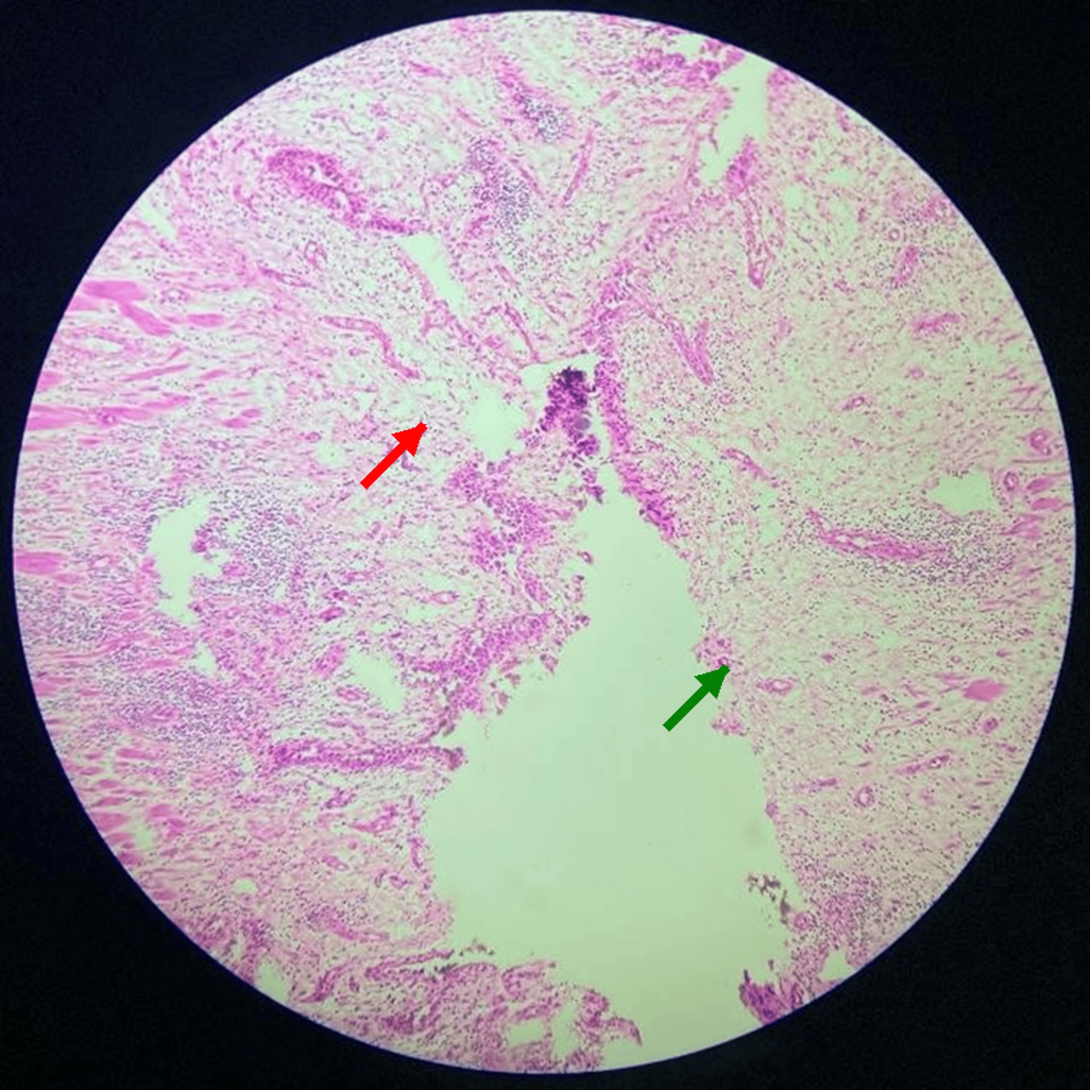
Histopathological examination of the resected distal ureter (H&E, 10× magnification) Histopathological examination reveals high-grade urothelial carcinoma with evident invasion into the lamina propria and muscularis propria. The tumor nests display pleomorphic hyperchromatic nuclei and loss of cellular polarity (green arrow). There is focal lymphovascular invasion (red arrow) with scattered inflammatory infiltrates in the surrounding stroma. No carcinoma in situ is noted in the adjacent urothelium. The surgical margins were free of tumor. H&E: Hematoxylin and eosin

Histopathology confirmed high-grade invasive UTUC (pT2) with lympho-vascular invasion (Figure 6), negative surgical margins, and one negative hilar lymph node. Four cycles of gemcitabine (1,000 mg/m² D1,8) plus cisplatin (70 mg/m² D1) were completed uneventfully as per established protocol [12]. Postoperative recovery was uncomplicated (Clavien-Dindo I ileus).

Follow-up protocol

Surveillance was tailored to the patient’s solitary kidney and high-risk urothelial carcinoma history. Clinical review, stoma inspection, and urine cytology were performed every three months during the first year and every six months thereafter. Multi-phase CT urography (arterial, nephrographic, and 8-minute excretory phases) was obtained at 3, 9, and 18 months using weight-based low-osmolality iodinated contrast (iohexol 350 mg I/mL, 1.5 mL/kg; maximum 120 mL). Conduit endoscopy is reserved for unexplained haematuria or positive cytology and has not been required to date. At 18 months the patient remains asymptomatic, stoma healthy, estimated glomerular filtration rate (eGFR) 76 mL/min/1.73 m², and imaging shows no recurrence.

## Discussion

Recurrent UTUC arising after radical cystectomy demonstrates more aggressive behavior and poorer cancer-specific survival than primary disease, mandating heightened clinical vigilance [2]. The present case reinforces the concept of pan-urothelial field cancerization, illustrating that patients with negative distal-ureter margins remain at lifelong risk. Conduit hematuria is frequently misattributed to stomal trauma or infection; however, it may be the sole external manifestation of an upper-tract recurrence. Multiphasic, high-resolution CT urography remains the most sensitive first-line modality, whereas MR urography offers comparable accuracy without nephrotoxic contrast or radiation, a distinct advantage in diverted systems [8]. Future surveillance algorithms are likely to incorporate circulating tumor DNA triggers alongside conventional imaging, as ctDNA can detect molecular relapse months before radiology [13].

Conservative measures for bleeding ileal conduit mucosa, such as topical silver nitrate, endoscopic laser fulguration, selective arterial embolization and radiotherapy, provide haemostasis but do not eradicate underlying malignancy, leading to repeated interventions. For localized high-grade UTUC, radical nephroureterectomy with bladder-cuff excision remains the oncologic gold standard. Long-term series demonstrate five-year cancer-specific survival of roughly 70% when surgery is complemented by cisplatin-based adjuvant chemotherapy [14]. Should systemic relapse occur, targeted agents such as infigratinib have produced objective responses in FGFR3-mutant tumors [15].

Uretero-enteric anastomotic configuration may influence both benign stricture formation and oncologic outcomes. Wallace conjoined anastomosis yields fewer strictures and shows a trend toward reduced recurrence compared with the Bricker technique on 25-year follow-up [16,17]. Although hydronephrosis and positive ureteric margin (but not anastomotic type) emerged as independent predictors of upper-tract recurrence in a recent meta-analysis, the left anastomosis was converted to Wallace type during salvage surgery to mitigate potential late complications [18].

Despite being uncommon, recurrent UTUC after radical cystectomy is being reported with increasing frequency. Table 2 juxtaposes the present case with pivotal series, emphasizing the survival benefit conferred by prompt surgical excision and gemcitabine-cisplatin (GC) adjuvant chemotherapy.

**Table 2:** Comparative outcomes of upper-tract recurrence following radical cystectomy GC: Gemcitabine-cisplatin; DFS: Disease-free survival; NED: No evidence of disease; UTUC: Upper-tract urothelial carcinoma

Study	Age/Sex	Anastomosis	Presentation	Management	Outcome
Current Case	48 / M	Bricker → Wallace (revised)	Isolated conduit hematuria	Laparoscopic nephroureterectomy + adjuvant GC	NED at 18 months
Stemrich et al. [11]	58 / M	Wallace	Hematuria + hydronephrosis	Robot-assisted nephroureterectomy + conduit revision	NED at 24 months
Ślusarczyk et al. [19]	62 / F	Bricker	Incidental CT lesion	Open salvage surgery	Local recurrence at 12 months
Birtle et al. [14]	Cohort (n = 261)	Mixed	Symptomatic/asymptomatic	Adjuvant GC vs surveillance	55% DFS benefit with GC
Krafft et al. [16]	Cohort (n = 178)	Bricker / Wallace	Anastomotic UTUC	Nephroureterectomy ± adjuvant therapy	Recurrence unrelated to anastomosis

Risk-adapted nomograms that integrate clinicopathological variables and molecular markers already outperform (Tumor-Node-Metastasis) TNM staging in predicting recurrence. Detecting asymptomatic relapses translates into improved cancer-specific survival, whereas timely symptom-triggered imaging, minimally invasive radical excision and evidence-based systemic therapy continue to deliver durable disease control.

Learning points

Conduit hematuria should never be dismissed. Prompt imaging may reveal curable upper-tract recurrence. Radical nephroureterectomy with selective conduit revision and adjuvant chemotherapy achieves durable control; choice of uretero-enteric anastomosis (Bricker versus Wallace) should balance stricture and potential recurrence risk. Innovations such as ctDNA guided surveillance promise more personalized follow up.

## Conclusions

Isolated distal-ureter recurrence after radical cystectomy is uncommon yet potentially lethal. In this case, prompt imaging of seemingly minor conduit bleeding uncovered a surgically curable UTUC. Laparoscopic nephroureterectomy with selective conduit revision preserved diversion integrity and renal function, while adjuvant GC secured durable oncological control. Vigilant symptom-triggered evaluation, oncologically sound salvage surgery, and guideline-based systemic therapy remain pivotal; prospective studies on anastomotic technique and liquid-biopsy surveillance are warranted to refine long-term management.
